# How Much Is Too Much? Exploring Pseudohyperaldosteronism in Glycyrrhizic Acid Toxicity From Chronic Licorice Root Consumption

**DOI:** 10.7759/cureus.16454

**Published:** 2021-07-18

**Authors:** Palak Patel, Mina Aknouk, Amanda Dawson, Ashley Aya, Anish Kanukuntla, Priyaranjan Kata, Anna De Dona

**Affiliations:** 1 Internal Medicine, Hackensack Meridian Health Ocean Medical Center, Brick, USA; 2 Internal Medicine, Hackensack Meridian Ocean Medical Center, Brick, USA

**Keywords:** licorice, pseudohypoaldosteronism, glycyrrhizic acid, hypokalemia, hypertension, 11-ß-hsd, mineralocorticoid receptor, aldosterone

## Abstract

Licorice has been around for centuries and has been commercialized in the food, tobacco, and healthcare industry. Historically, its therapeutic benefits have been reaped in countless ways, including as a thirst sensation suppressor in battlefields, flavoring agent in medicinal preparations, antacid for gastric discomfort and peptic ulcers, and even as an estrogenic agent in postmenopausal women. Licorice and its derivatives are recognized safe by the US Food and Drug Administration (FDA). Though FDA recognized the licorice to be a food additive in certain concentrations, it has issued warnings against its use in at-risk group and in larger amount. However, it is a lesser known fact that glycyrrhizic acid, the active component in licorice, can cause a metabolic syndrome presenting as pseudohyperaldosteronism. Chronic consumption leads to the development of hypertension, metabolic alkalosis, and hypokalemia. We present a patient who developed a sinus pause on telemetry and subsequent syncope after presenting for evaluation of hypertension and hypokalemia. The patient had been ingesting a significant quantity of deglycyrrhizinated licorice for many years to alleviate postprandial epigastric pain. Although seemingly benign electrolyte disturbance, it is crucial to recognize that chronic consumption of licorice without strict regulation can lead to supraventricular and ventricular ectopics and tachyarrhythmias with the potential to develop life-threatening arrhythmias including ventricular tachycardia, ventricular fibrillation, and Torsades de Pointes.

## Introduction

Glycyrrhizic acid, a component of licorice extract, has long been utilized as a natural sweetener and thirst reliever. Many people who ingest big amounts of it are unaware of the negative side effects and tend to overestimate its medicinal benefits. Licorice is a dietary supplement that has been approved by the US Food and Drug Administration (FDA) and is widely used in a variety of products with no strict distribution or consumption regulations. The active metabolite in licorice, glycyrrhetic acid, inhibits the enzyme 11-ß-hydroxysteroid dehydrogenase enzyme type 2, resulting in a cortisol-induced mineralocorticoid reaction and an inclination toward sodium elevation and potassium reduction [1]. The fact that it has aldosterone-like properties is crucial in understanding both its health benefits and its wide range of adverse effects. Despite its medicinal efficacy in providing mild relief and the treatment of gastric indigestion, persistent licorice use can lead to serious and even life-threatening consequences. In this case, we present a patient with apparent mineralocorticoid overload with subsequent development of sinus pause and syncope due to severe hypokalemia secondary to chronic licorice consumption.

## Case presentation

A 61-year-old male was referred to the hospital by his primary care physician for further evaluation of low potassium levels and elevated blood pressure. He initially went to his physician because he had experienced bilateral leg swelling for a week. Past medical history was significant for hypertension and fibromyalgia. Family history was significant for cardiovascular disease. He denied any alcohol, tobacco, or recreational drug consumption.

**Figure 1:**
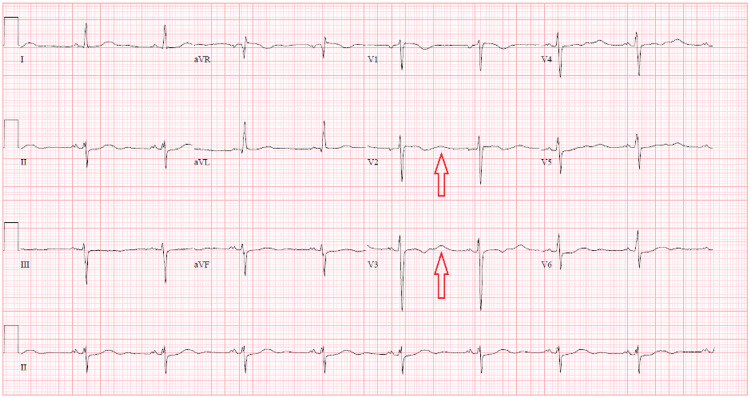
EKG with normal sinus rhythm, flattened T waves and U waves

In the emergency department (ED), his vital signs were temperature 98.2 F, blood pressure 228/115 mmHg, pulse 80 beats per minute, respiratory rate 18 per minute, and SpO_2_ 99%. On physical examination, abdomen was soft, bowel sounds were present with mild epigastric tenderness to palpation, and 1+ pitting edema on bilateral lower extremities. His laboratory result showed hemoglobin of 14.3 g/dL (13.2-17.5 g/dL), white blood cell count of 6.3 x 10^3^/uL (4.5-11.0 x 10^3^/uL), and platelet count of 201 x 10^3^/uL (140-450 x 10^3^/uL). Comprehensive metabolic panel showed a blood urea nitrogen of 18 mg/dL (5-25 mg/dL), serum creatinine of 0.96 mg/dL (0.61-1.24 mg/dL), sodium of 139 mmol/L (136-145 mmol/L), potassium of 2.1 mmol/L (3.5-5.2 mmol/L), chloride of 91 mmol/L (96-110 mmol/L), bicarbonate of 38 mmol/L (24-31 mmol/L), glucose of 89 mg/dL (70-99 mg/dL), alanine aminotransferase of 21 U/L (10-60 U/L), aspartate aminotransferase of 27 U/L (10-42 U/L), alkaline phosphatase of 89 U/L (38-126 U/L), and a total bilirubin of 1.1 mg/dL (0.2-1.3 mg/dL). EKG showed normal sinus rhythm with a rate of 60 beats per minute with flattened T-wave and presence of the U-wave complexes (Figure 1). Labetolol HCl 20 mg IV and hydralazine 10 mg IV were given for blood pressure control in the ED as well as potassium 40 mEq PO and protonix 40 mg PO.

Once admitted and upon further questioning, the patient revealed that he was experiencing abdominal pain for the past 15 years. He describes himself as a "naturalist" and denies taking the proton-pump inhibitors that were initially prescribed to him. He wanted to resolve his abdominal pain with a natural remedy and resorted to over-the-counter deglycyrrhizinated licorice supplements for pain relief after multiple dietary variations had failed. Over the years, he had increased his daily dose for postprandial pain relief. He admitted that for the past 6-8 months he was consuming up to 20 licorice root 760 mg tablets a day, well exceeding the acceptable daily allowance (2280 mg). He further revealed he had a 10-pound weight loss in two months due to decreased oral intake. The last colonoscopy was over 10 years ago. The abdominal pain associated with oral intake was the reason he refused to take oral medications including home medication losartan-hydrochlorothiazide 100-12.5 mg and potassium PO repletion prescribed to him from the ED. Medication formulations were switched to IV.

A rapid response was called after the patient had a 10-second pause on telemetry. Repeated potassium levels showed potassium of 1.9 mmol/L and the patient was brought to the critical care unit for close observation. The patient was educated and was willing to receive potassium via oral solution in addition to the potassium he was receiving intravenously. The patient’s potassium levels were monitored every 4 hours as well as serial EKGs; he was unable to achieve a potassium level higher than 2.8 mmol/L in 24 hours. The potassium was eventually normalized to 3.5 mmol/L 48 hours post-admission via continued potassium IV infusion and oral intake equalling to 120 mEq daily. 

Gastroenterology was consulted and an endoscopy was done, which showed gastritis and gastroesophageal reflux disease without evidence of *Helicobacter pylori* or other pathology. He was started on sucralfate 1 g suspension, reglan 10 mg, and continued on pantoprazole 40 mg IV. Psychiatry was also consulted; he was willing to take oral medications and was started on sertraline 25 mg QD and buspirone 10 mg TID. Carvedilol 12.5 BID, irbesartan 300 mg daily, and spironolactone 25 mg BID were all prescribed.

Systolic blood pressure remained above 200 despite several days on the antihypertensive medications including hydralazine 10 mg IV PRN. Despite dose adjustments of the antihypertensive medications, the patient’s blood pressure remained labile for over one week. Secondary causes of hypertension were investigated. Renin and aldosterone study showed plasma renin <0.1 ng/mL/h (0.2-1.6 ng/mL/h ), aldosterone level <3 ng/dL (<16 ng/dL), serum cortisol level 22 μg/dL (8.7-22.4 μg/dL), and 11 deoxy-cortisol <5 ng/dL (<19 ng/dL). Plasma metanephrines 0.22 nmol/L (0-0.49 nmol/L) and urine metanephrines of 122 μg/d (55-320 μg/d) were also unremarkable. Renal ultrasound and CT without contrast of the abdomen and pelvis were non-significant. Amlodipine 5 mg QD was added. Prior to discharge, the patient’s systolic blood pressure ranged between 120 and 160. He was sent home with a blood pressure cuff. The patient was thoroughly re-educated on the importance of medication compliance and the cessation of licorice supplement intake.

## Discussion

Licorice is derived from the plant *Glycyrrhiza glabra*, with its Greek origin glykos, meaning sweet, and rhiza, which translates to root. Its therapeutic or other commercial use dates back to the Egyptians and Assyrians in the first millennium BC. Licorice-containing products are used in a wide range of products, including confectioneries, health products, chewing tobacco, chewing gum, and some alcoholic drinks [2]. In the United States, it is recognized as a safe flavoring agent and is approved as a safer natural product in many other countries. Many people are uninformed of its negative consequences and mistake it for a benign low-fat snack or a nutritious food choice [3]. According to the World Health Organization (WHO), consumption of 100 mg a day is unlikely to cause side effects.

Glycyrrhizin (GL) is a triterpenoid compound that gives licorice root its sweet flavor. It is made up of potassium-calcium-magnesium salts of glycyrrhizic acid. The amount of GL found in licorice roots ranges from 2% to 25% depending on the plant. GL has a 50-fold sweeter taste than sucrose (cane sugar) [4].

In humans, the main component of licorice, glycyrrhizic acid, is hydrolyzed (pre-systemic hydrolysis) to glycyrrhetinic acid by intestinal bacteria that have a specialized ß-glucuronidase enzyme [5]. Glycyrrhetinic acid is quickly absorbed and transported to the liver via carrier molecules. It is metabolized in the liver to glucuronide and sulfate conjugates, which are effectively transferred and excreted through the bile, where they are first exposed to entero-hepatic circulation, potentially resulting in sustained preservation of pharmacologically active plasma amounts [6]. The amount of glycyrrhetinic acid conjugates reabsorbed is largely determined by the movement rate of gastrointestinal contents through the small and large intestines. As a result, glycyrrhetinic acid can accumulate in subjects with long gastrointestinal transit periods, resulting in toxicity after repeated ingestion [4].

Glycyrrhetinic acid, which is an active metabolite of licorice extract, may cause a condition known as apparent mineralocorticoid overload [5]. These side effects are caused by the inhibition of the enzyme 11-ß-HSD, which is responsible for renal conversion of cortisol to inactive cortisone, thus leading to an increased amount of cortisol [4]. This result is physiologically significant because cortisol binds to the mineralocorticoid receptor with the same strength as aldosterone [6].

**Figure 2:**
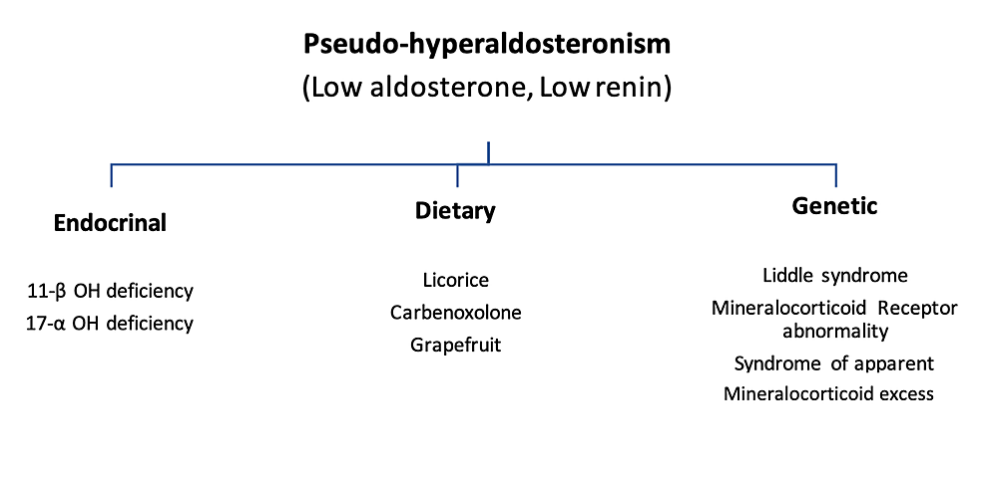
Different causes of pseudo-hyperaldosteronism

Primary, secondary, and pseudo-hyperaldosteronism are the three forms of hyperaldosteronism [4]. Pseudo-hyperaldosteronism is a syndrome in which plasma renin production and aldosterone levels are suppressed, mimicking hyperaldosteronism clinically (Figure 2). In patients with unexplained hypokalemia and muscle fatigue, licorice overconsumption should be suspected clinically especially in the appropriate clinical setting with a significant dietary history. The inhibition of 11-ß-HSD by licorice results in a decrease in cortisol to cortisone conversion. As a result, the cortisol:cortisone level in peripheral venous plasma rises dramatically. 

Our patient who initially presented with uncontrolled hypertension and subsequently developed syncope displayed cardiovascular manifestations. There are various complications related to chronic licorice consumption. Table 1 demonstrates the licorice-related risks that have been historically mentioned [4]. 

**Table 1:** Clinical manifestations of licorice toxicity CPK: creatine phosphokinase; CYP3A4: cytochrome P450 3A4

System	Clinical Manifestations
Cardiovascular	Hypertension, hypertensive encephalopathy, cardiac arrhythmias, and death due to QT prolongation, heart failure and pulmonary edema, Generalized edema, embolic ischemia
Electrolyte and renal abnormalities	Hypokalemia, metabolic alkalosis, elevated CPK, acute tubular necrosis due to myoglobinuria
Neurological	Hypokalemic myopathy, stroke, rhabdomyolysis, carpal tunnel syndrome, licorice-induced myoclonus, ocular deficits
Allergic reactions	Occupational asthma, contact dermatitis
Drug interaction	Inhibition of P450 and CYP3A4 systems, potentiation of the effect of warfarin therapy, digoxin toxicity due to licorice-induced hypokalemia

The licorice-induced mineralocorticoid excess is reversible; however, the associated suppressed enzyme processes have different rates of recovery. Since licorice was withdrawn, 11-hydroxysteroid dehydrogenase activity was inhibited for two weeks. The renin-aldosterone system's basal production, on the other hand, remained poor for several months after licorice withdrawal. It can be treated with the cessation of licorice consumption and potassium replacement. Potassium-sparing diuretic, spironolactone, and dexamethasone should be consumed.

## Conclusions

The potency of licorice toxicity is shown by the sustained inhibition of the renin-aldosterone axis, emphasizing the importance of considering licorice, as well as other causes or medications that impair 11-hydroxysteroid dehydrogenase function, as a source of low-renin hypertension. In this case, we have a 61-year-old male with uncontrolled hypertension and hypokalemia who developed syncope secondary to severe hypokalemia in the setting of chronic licorice consumption. Every physician treating a patient of peptic ulcer should explore possibility of use of licorice by the patient and It is essential to realize that while natural herbal supplements may seem benign and provide mild health benefits and aid discomfort, excessive or chronic consumption is detrimental. As the common phrase goes, “too much of anything is never good!”
